# Efficacy of three sputum specimens for the diagnosis of *Mycobacterium avium* complex pulmonary disease

**DOI:** 10.1186/s12890-023-02327-5

**Published:** 2023-01-19

**Authors:** Naohisa Urabe, Susumu Sakamoto, Marie Masuoka, Chiaki Kato, Asuka Yamaguchi, Nozomi Tokita, Sakae Homma, Kazuma Kishi

**Affiliations:** Department of Respiratory Medicine, Omori Medical Center, Toho University, 6-11-1 Omori-nishi, Ota-ku, Tokyo, 143-8541 Japan

**Keywords:** *Nontuberculous mycobacterium*, *Mycobacterium avium* complex, Sputum specimens, Diagnosis, Anti-glycopeptidolipid-core IgA antibody

## Abstract

**Background:**

In *Mycobacterium avium* complex pulmonary disease (MAC-PD), diagnosis requires a positive culture from at least two separate expectorated sputum specimens. The optimal number of sputum examinations remains unclear.

**Objective:**

This study sought to elucidate the diagnostic yield of acid-fast bacilli in MAC-PD using 3 sputum specimens and to clarify the clinical characteristics of patients with MAC-PD diagnosed using 3 sputum specimens. Furthermore, we investigated the correlation between increased number of sputum specimens and diagnostic yield.

**Methods:**

We reviewed the medical records of 139 patients with MAC-PD diagnosed at Toho University Omori Medical Center for whom at least three sputum specimens were examined before treatment from November 2014 through June 2021. Patients were classified into the 3-sputum diagnosed and the non-3 sputum diagnosed groups based on diagnostic procedure; clinical and radiological characteristics were compared. We also assessed diagnostic yield with the increased number of sputum specimens.

**Results:**

Diagnostic yield with 3 sputum specimens was 16.5% (23/139). The 3-sputum diagnosed group had a lower body mass index [18.6(17–19.5) vs. 19.5(18–21.5); *p* = 0.014], and higher chest CT score [9(6.5–13) vs. 6(4–9); *p* = 0.011] including cavitary lesions (39.1% vs. 19%; *p* = 0.037) compared with the non-3 sputum diagnosed group. When the number of sputum specimens was increased to 6, the diagnostic yield increased to 23.7% (33/139).

**Conclusion:**

Diagnostic yield with 3 sputum specimens was 16.5%. Patients diagnosed using 3 sputum specimens had more severe chest CT findings including cavitary lesions. Increasing the number of sputum specimens to 6 improved diagnostic yield by 7.2%.

**Supplementary Information:**

The online version contains supplementary material available at 10.1186/s12890-023-02327-5.

## Background

*Nontuberculous mycobacterial* pulmonary disease (NTM-PD) is increasing in incidence worldwide and has become an important concern [1]. Likewise, the incidence of NTM-PD is gradually increasing in Japan and exceeded the incidence of *Mycobacterium tuberculosis* (*M. tuberculosis*) infection for the first time in 2014 [2]. The NTM type differs by region. In Japan, *Mycobacterium avium* complex (MAC) accounts for about 90% of the total [2]. According to the current American Thoracic Society/European Respiratory Society/European Society of Clinical Microbiology/Infectious Disease Society of America (ATS/ERS/ESCMID/IDSA) guidelines, a diagnosis of MAC pulmonary disease (MAC-PD) requires a positive culture from at least 2 separate expectorated sputum specimens or at least 1 bronchial lavage sample as well as pulmonary symptoms and chest computed tomography (CT) findings consistent with MAC-PD [3]. This is based on a study where 98% of patients with ≥ 2 sputum cultures had clinically significant MAC-PD [4]. Also, 3 sputum specimens are recommended for the diagnosis of pulmonary *tuberculosis* (PTB) [5]. In Japan, 3 sputum specimens are also customarily used for the diagnosis of MAC-PD. Although PTB can be definitively diagnosed based on single culture-positive or PCR-positive results, the diagnosis of MAC-PD requires 2 positive cultures. However, the optimal number of sputum specimens to obtain 2 positive cultures is unclear. This study aimed to clarify the diagnostic yield of acid-fast bacilli (AFB) for MAC-PD using 3 sputum specimens and the clinical characteristics of the patients leading to the diagnosis. We also assessed the diagnostic yield with an increased number of sputum specimens. Furthermore, we proposed new criteria to improve the diagnostic yield while maintaining the quality of diagnosis.

## Methods

### Study design

**Fig. 1:**
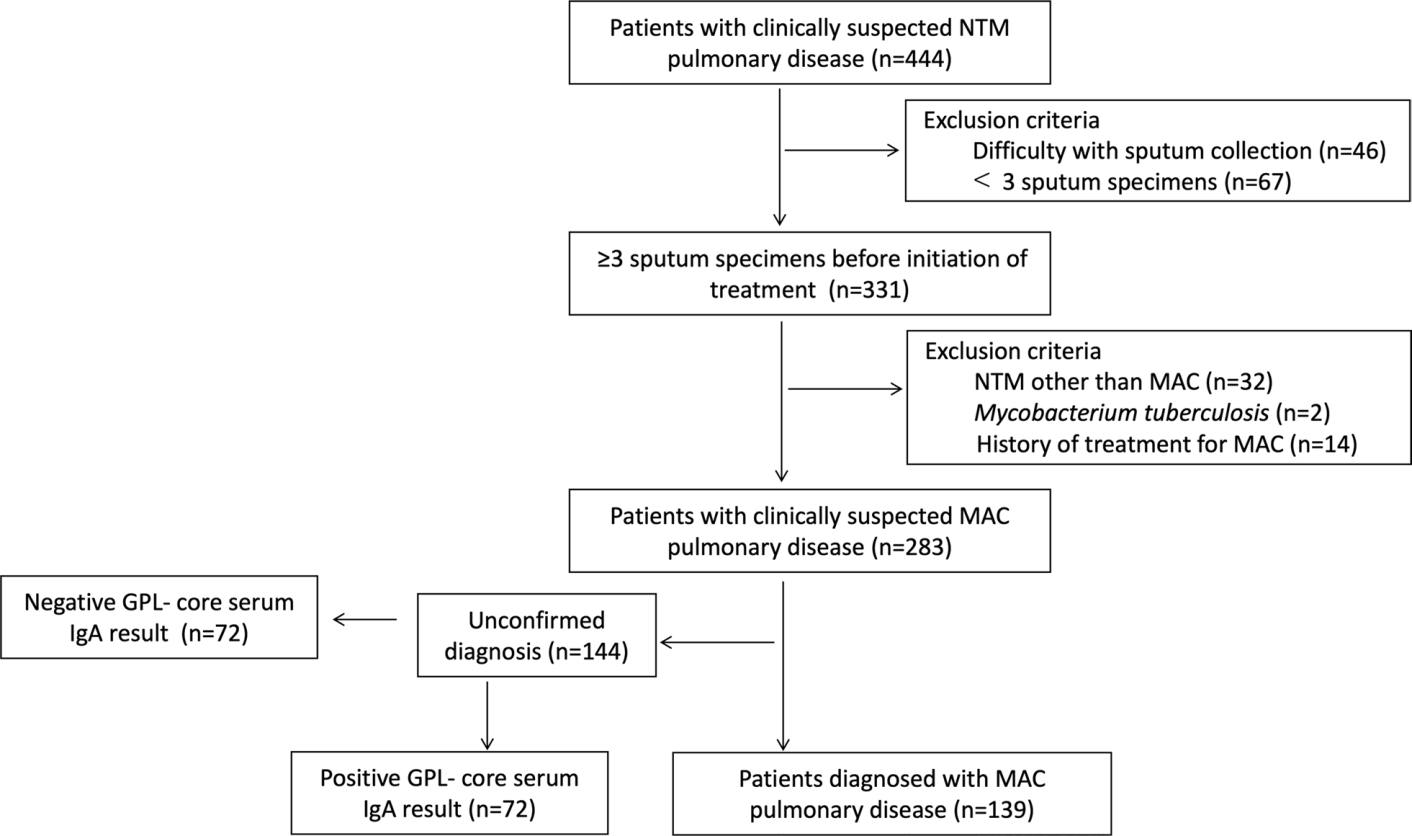
Flowchart showing the study design between November 2014 and June 2021. NTM, *Nontuberculous mycobacteria*; MAC, *Mycobacterium avium* complex; GPL, Glycopeptidolipid

This single-center retrospective cohort study included 444 patients with suspected NTM-PD on chest CT findings, seen at Toho University Omori Medical Center from November 2014 through June 2021. We excluded those patients who had difficulty with sputum collection, those for whom less than 3 sputum specimens were collected before treatment for MAC, and who had diagnoses of mycobacterium tuberculosis and NTM other than MAC, and history of treatment for MAC. Of the remaining 283 patients with clinically suspected MAC-PD, 139 patients who satisfied the ATS/ERS/ESCMID/IDSA guidelines diagnostic criteria for MAC-PD were enrolled (Fig. 1). Sputum specimen induction was not performed. The interval between collection of the 3 sputum specimens differed for each patient, but the shortest interval from the first sputum specimen to the third was 2 days; the longest was 3 months.

Based on the examination of 3 sputum specimens, the 139 patients were classified into the 3 sputum specimens diagnosed group (3-sputum diagnosed group) and non-3 sputum specimens diagnosed group (non-3 sputum diagnosed group); their clinical and radiological characteristics were compared. Univariate and multivariate logistic regression analyses were performed to identify factors independently associated with a diagnosis of MAC-PD using 3 sputum specimens. We assessed the diagnostic yield when the number of sputum specimens was increased.

Additionally, we ascertained whether the new diagnostic criteria for MAC-PD proposed by Kawasaki et al. [6] would improve diagnostic yield in 3 sputum specimens as follows, “ ≥ 1 positive sputum culture and GPL-core serum IgA positivity”.

### Data collection

We collected data on age, sex, body mass index (BMI), smoking history, serum anti-glycopeptidolipid (GPL)-core IgA titer, comorbidities, chest and paranasal sinuses CT, and sputum and bronchoscopy culture results. Overall, 118 of 139 (84.9%) patients underwent chronic obstructive pulmonary disease (COPD) assessment testing (CAT) to confirm subjective symptoms at the time of diagnosis [7].

### Microbiological examination

Sputum specimens were subjected to Ziehl–Neelsen and Gram staining and cultured for mycobacteria, other bacteria, and fungi. Mycobacteria culture was carried out using a 2% Ogawa egg medium (Kyokuto Pharmaceutical Industrial Co., Tokyo, Japan). PCR assays were performed for *M. tuberculosis*, *M. avium*, and *M. intracellulare*. We used DNA–DNA hybridization to identify non-MAC species.

### Chest CT score

Chest high-resolution computed tomography (HRCT) score was reviewed as previously described [8, 9]. We divided the lungs into 6 zones at the levels of the carina and inferior pulmonary vein. We then categorized the 4 types of pulmonary lesions in MAC [cavities, bronchiectasis, nodules (less than 10 mm), and infiltration (an area of opacity larger than 10 mm)] into 5 stages, according to the occupation area in each Sect. (0: no lesion, 1: 1% to 24% occupied, 2: 25% to 49% occupied, 3: 50% to 74% occupied, and 4: 75% to 100% occupied). Two respiratory physicians with over 15 years of experience reviewed the chest CT scores independently.

### Glycopeptidolipid core serum IgA assay

Levels of serum IgA antibody against the GPL-core antigen of MAC were measured using an enzyme immunoassay kit (TAUNS Laboratory Inc., Shizuoka, Japan). This kit is used to measure antibody levels via an enzyme-linked immunosorbent assay. GPL-core IgA antibody was developed by Kitada et al. [10] and has been commercially available in Japan since 2011.

### Statistical analysis

Data are presented as the number of patients and percentages. Age, BMI, chest CT score, and CAT score are expressed as median values (with interquartile range). Associations of categorical and continuous variables between patients in the 3-sputum diagnosed group and non-3 sputum diagnosed group were tested with the Chi-squared or Fisher’s exact test, and the Mann–Whitney U test, respectively. Univariate and multivariate logistic regression were used to evaluate factors independently associated with a definitive diagnosis. Multivariate logistic regression was performed using a stepwise method. A *p* value of < 0.05 was considered to indicate statistical significance. Statistical analyses were performed with SPSS software version 27 (IBM Corp., Armonk, NY).

### Ethics

The study protocol was approved by the Ethics Committee of Toho University Omori Medical Center (Approval No. M21239). All experiments were conducted in accordance with the Declaration of Helsinki guidelines for studies involving human participants (2008).

## Results

### Clinical characteristics

This study included 139 patients; the 3-sputum diagnosed group comprised 23 (16.5%) patients (5 men, 18 women; median age 73 years) and the non-3 sputum diagnosed group included 116 patients (17 men, 99 women; median age 70 years). Table 1 shows the characteristics of all 139 patients. The 3-sputum diagnosed and non-3 sputum diagnosed groups showed significant differences in BMI [18.6(17–19.5) vs. 19.5(18–21.5); *p* = 0.014], and subjective cough symptoms in CAT score [2.5(1–3) vs. 1(0–2); *p* = 0.038]. Both groups differed significantly in chest CT scores [9(6.5–13) vs. 6(4–9); *p* = 0.011], and the proportion of patients with cavitary lesions (39.1% vs. 19%; *p* = 0.037). Table 2 shows the results of microbiological analysis. The proportion of patients with ≥ 2 out of 3 positive sputum MAC-PCR and smear tests was significantly higher in the 3-sputum diagnosed group [(87% vs. 12.1%; *p* < 0.001), (26.1% vs. 1.7%; *p* < 0.001)]. Bronchoscopy was performed significantly more frequently in the non-3 sputum diagnosed group (47.8% vs. 87.9%; *p* = 0.049).

**Table 1:** Clinical characteristics of patients in the 3-sputum diagnosed and non-3 sputum diagnosed groups

Characteristic	Total	3 sputum diagnosed group	non-3 sputum diagnosed group	*p* value
No. of patients	139	23	116	
Age: (years); median (range)^a^	70 (62–75)	73 (63–78)	70 (61–75)	0.333
Sex: female; n (%)	117 (84.2)	18 (78.3)	99 (85.3)	0.366
BMI: (kg/m^2^); median (range)^a^	19.3 (17.5–21.3)	18.6 (17–19.5)	19.5 (18–21.5)	**0.014**
Smoking: never; n (%)	107 (77)	16 (69.6)	91 (78.4)	0.417
Positive GPL-core serum IgA result; n (%)	99 (71.2)	17 (73.9)	82 (70.7)	1.000
Value of GPL-core serum IgA (U/mL); (range)^a^	3.0 (1.2–7.4)	5.4 (2.4–8.9)	2.5 (1.2–6.7)	0.253
Comorbidities: n (%)				
Rheumatoid arthritis (RA)	18 (12.9)	2 (8.7)	16 (13.8)	0.737
Sinusitis	17 (12.2)	2 (8.7)	15 (12.9)	0.738
Underlying pulmonary disease: n (%)				
Emphysema	5 (3.6)	1 (4.3)	4 (3.4)	1.000
Interstitial pneumonia	13 (9.4)	2 (8.7)	11 (9.5)	1.000
Concomitant drug; n (%)				
Corticosteroids	12 (8.6)	3 (13)	9 (7.8)	0.419
Immunosuppressant	12 (8.6)	2 (8.7)	10 (8.6)	1.000
Biopharmaceutical	6 (4.3)	1 (4.3)	5 (4.3)	1.000
Erythromycin	8 (5.8)	1 (4.3)	7 (6)	1.000
Infective MAC strain; n (%)				
* M. avium*	114 (82)	19 (82.6)	95 (81.9)	1.000
* M. intracellulare*	28 (20.1)	4 (17.4)	24 (20.7)	1.000
Co-infection n (%)				
* P. aeruginosa*	8 (5.8)	2 (8.7)	6 (5.2)	0.619
* H. influenzae*	6 (4.3)	1 (4.3)	5 (4.3)	1.000
MSSA	15 (10.8)	1 (4.3)	14 (12.1)	0.465
* Aspergillus* spp.	2 (1.4)	0	2 (1.7)	1.000
Subjective symptoms (range)^a^				
CAT score	6.5 (3.3–12)	10.0 (7–16.3)	6 (3–11)	0.075
Cough score ^b^	1 (1–2)	2.5 (1–3)	1 (0–2)	**0.038**
Sputum score^b^	1 (0–2)	2 (1–3)	1 (0–2)	0.158
Chest CT score				
Total chest CT score; median (range)^a^	7 (4–10)	9 (6.5–13)	6 (4–9)	**0.011**
Nodular lesion score in chest CT score	3 (2–6)	5 (2–8)	3 (2–5)	0.084
Infiltration score in chest CT score	1 (0–2)	1 (1–2)	1 (0–2)	0.155
Bronchiectasis score in chest CT score	2 (1–3)	2 (1–3)	2 (1–3)	0.612
Cavitary lesion score in chest CT score	0 (0–0)	0 (0–1)	0 (0–0)	0.069
Cavitary lesion; n (%)	31 (22.3)	9 (39.1)	22 (19)	**0.037**
Radiographic pattern; n (%)		2 (1–3)		
Non-cavitary NBE type	108 (77.7)	14 (60.9)	94 (81)	0.052
FC type	4 (2.9)	2 (8.7)	2 (1.7)	0.128
Cavitary NBE type	27 (19.4)	7 (30.4)	20 (17.2)	0.156

BMI, Body mass index; GPL, Glycopeptidolipid; MAC, *Mycobacterium avium* complex; *P.aeruginosa, Pseudomonas aeruginosa*; *H. influenzae*, *Haemophilus influenzae*; MSSA, Methicillin-sensitive *staphylococcus aureus*; CAT, COPD assessment test; CT, Computed tomography; NBE, Nodular bronchiectatic; FC, Fibrocavitary; *P *value < 0.05 were shown in boldface

^a^Interquartile range; ^b^ Score for subjective symptoms of cough and sputum, included in CAT score

**Table 2:** Sputum specimens and bronchoscopy culture results of patients in the 3-sputum diagnosed and non-3 sputum diagnosed groups

Characteristic	Total	3-sputum diagnosed group	Non-3 sputum diagnosed group	*p* value
No. of patients	139	23	116	
Number of positive sputum cultures; n (%)				
0	80 (57.6)	0 (0)	80 (69.0)	
1	36 (25.9)	0 (0)	36 (31.0)	
≥ 2	23 (16.5)	23 (100)	0 (0)	
Number of positive sputum MAC-PCR; n (%)				
0	70 (50.4)	0	70 (60.3)	**< 0.001**
1	35 (25.2)	3 (13.0)	32 (27.6)	0.191
≥ 2	34 (24.4)	20 (87.0)	14 (12.1)	**< 0.001**
Number of positive sputum smear; n (%)				
0	112 (80.6)	10 (43.5)	102 (87.9)	**< 0.001**
1	19 (13.7)	7 (30.4)	12 (10.3)	**0.018**
≥ 2	8 (5.8)	6 (26.1)	2 (1.7)	**< 0.001**
Underwent bronchoscopy; n (%)	113 (81.3)	11 (47.8)	102 (87.9)	**0.049**
Positive culture for MAC with bronchoscopy; n (%)	109/113 (96.5)	11/11 (100)	98/102 (96.1)	0.812

MAC, *Mycobacterium avium* complex; *P* values < 0.05 were shown in boldface

### Factors independently associated with diagnosis using 3 sputum specimens

Table 3 shows the results of univariate and multivariate logistic regression analysis of independent associations with diagnosis in 3 sputum specimens. Lower BMI (Odds Ratio [OR], 0.83; 95% confidence interval [CI], 0.7–0.99; *p* = 0.039), cavitary lesion (OR, 2.75; 95% CI, 1.05–7.16; *p* = 0.039) higher chest CT score (OR, 1.17; 95% CI, 1.05–1.31; *p* = 0.004), higher cough score in CAT score (OR, 1.52; 95% CI, 1.06–2.16; *p* = 0.021) were independently associated with diagnosis in 3 sputum specimens in univariate analysis. Lower BMI (OR, 0.76; 95% CI, 0.59–0.99; *p* = 0.039) and higher chest CT score (OR, 1.22; 95% CI, 1.04–1.44; *p* = 0.018) were independently associated with diagnosis in 3 sputum specimens in multivariate analysis.

**Table 3:** Univariate and multivariate logistic regression analysis of independent associations with diagnosis using 3 sputum specimens

Variable	Univariate logistic regression			Multivariate logistic regression		
	OR	95% CI	*p* value	OR	95% CI	*p* value
BMI	0.83	0.70–0.99	**0.039**	0.76	0.59–0.99	**0.039**
Cavitary lesion	2.75	1.05–7.16	**0.039**	3.41	0.96–12.07	0.057
Chest CT score	1.17	1.05–1.31	**0.004**	1.22	1.04–1.44	**0.018**
Cough score in CAT score	1.52	1.06–2.16	**0.021**	1.11	0.71–1.74	0.646

*BMI* Body Mass Index, *CAT* COPD assessment test, *MAC* *Mycobacterium avium* complex;* P* values < 0.05 were shown in boldface

### Change in diagnosed yield with increased number of sputum specimens

Table 4 shows the change in diagnostic yield with increased number of sputum specimens. There were 73, 53, 41, 29, 24, 21, and 16 patients for whom sputum specimens were obtained 4, 5, 6, 7, 8, 9, and 10 times before initiation of treatment for MAC. The cumulative diagnostic yield for MAC-PD was 18% (25/139), 21.6% (30/139), 23.7% (33/139), 23.7% (33/139), 24.5% (34/139), 25.2% (35/139), and 25.2% (35/139), respectively. Median intervals between collecting the 3rd sputum specimen to the 4th, 5th, 6th, 7th, 8th, 9th, and 10th sputum specimens were 4 (1–9), 9 (4.8–16), 15 (5.8–24), 14 (10–24), 17 (11–27), 20 (12–32) and 21 (17–27.5) months, respectively.

**Table 4:** Cumulative diagnostic yield for MAC-PD with increased number of sputum specimens

Number	Median interval^a^; month(range)^b^	No. of patients	Diagnosis: n	Cumulative rate of diagnosis (%)
3		139	23	16.5
4	4 (1–9)	73	2	18.0
5	9 (4.8–16)	53	5	21.6
6	15 (5.8–24)	41	3	23.7
7	14 (10–24)	29	0	23.7
8	17 (11–27)	24	1	24.5
9	20 (12–32)	21	1	25.2
10	21 (17–27.5)	16	0	25.2

*MAC-PD* *Mycobacterium avium* complex pulmonary disease

^a^Median interval between sputum specimen after 3 sputum specimens; ^b^ interquartile range

### Validation of new diagnostic criteria proposed by Kawasaki et al. for the diagnosis of MAC-PD

**Fig. 2:**
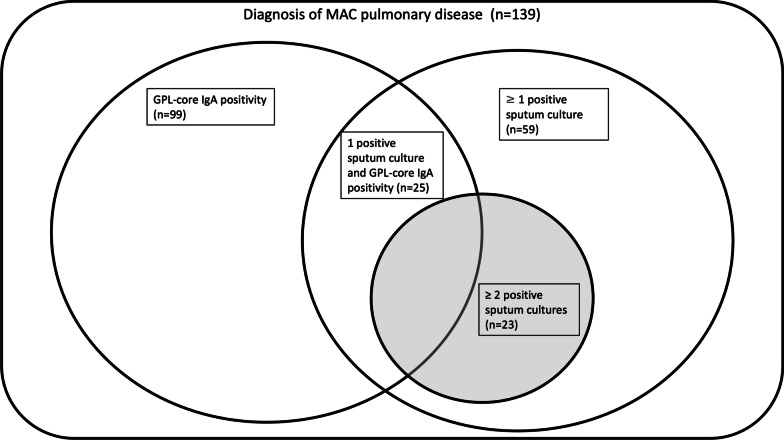
Distribution of diagnosis of MAC-PD patients as illustrated using a Venn diagram. Gray area represents patient with confirmed diagnosis of MAC-PD using 3 sputum specimens. MAC, *Mycobacterium avium* complex; GPL, Glycopeptidolipid

Figure 2 shows the distribution of diagnosis for patients with MAC-PD illustrated using a Venn diagram. Among the 139 MAC-PD patients, the 3-sputum diagnosed group (≥ 2 out of 3 positive sputum cultures), ≥ 1 out of 3 positive sputum cultures, and positive results for GPL-core serum IgA comprised 23 (16.5%), 59 (42.4%), and 99 (71.2%), respectively. Patients with 1 out of 3 positive sputum cultures and GPL-core serum IgA positivity comprised 25 (18%). According to the new diagnostic criteria of “ ≥ 1 positive sputum culture and GPL-core serum IgA positivity” proposed by Kawasaki et al., now added to the conventional ATS/ERS/ESCMID/IDSA criteria [3], the diagnostic yield was 34.5% (48/139) using 3 sputum specimens.

## Discussion

The diagnostic yield for MAC-PD using 3 sputum specimens was 16.5%. In MAC-PD, 3 sputum specimens are insufficient for definitive diagnosis. No study has yet investigated the number of sputum specimens required for a definitive diagnosis of MAC-PD, however, there have been studies in PTB. For instance, the cumulative positive proportion of AFB in induced sputum specimens of patients with active PTB from smear, PCR, and culture analysis was 64%, 89%, and 70% for 1, 81%, 95%, and 91% for 2, 91%, 99%, and 99% for 3, and 98%, 100%, and 100% for 4 sample inductions [11]. Repeated sputum specimens could considerably improve diagnostic accuracy for PTB. The ATS/Centers for Disease Control and Prevention/IDSA statement of 2017, strongly recommended 3 sputum specimens [5]. Because *M. tuberculosis* is not an environmentally endemic pathogen, even a single culture-positive or PCR-positive result suffices to establish a definitive diagnosis; MAC, however, has been isolated from environmental sources, including water and soil [12, 13]. Therefore, ≥ 2 positive cultures from sputum specimens are required for the diagnosis of MAC-PD considering the possibility of contamination from the environment or colonization. In Japan, conventionally 3 sputum specimens are collected for MAC-PD but in this study, this can be considered insufficient for diagnosis. Furthermore, the actual diagnostic yield of 3 sputum specimens may even be lower, because among the 72 patients with GPL-core serum IgA positivity with no definitive diagnosis in this study, it cannot be excluded that bronchoscopy may contribute to the diagnosis of MAC-PD. Moreover, the 72 patients positive for GPL-core serum IgA and with no definitive diagnosis had a significantly lower rate of undergoing bronchoscopy than the 139 patients with a definitive diagnosis (Additional file 1: Table S1).

Patients diagnosed using 3 sputum specimens had severe findings on chest CT, including cavitary lesions, severe subjective symptoms, and low BMI. Two previous studies compared patients diagnosed using sputum specimens with those diagnosed using bronchoscopy. Tamura et al. reported a higher proportion of patients diagnosed using sputum specimens among patients with cavitary lesions than those without (67% vs. 43%); there was no significant difference [14]. In a study by Maekawa et al., patients diagnosed using sputum specimens were significantly more symptomatic and had more widespread lesions, but there was no difference in the presence of cavities [15]. The characteristics of the patients in the previous reports are similar to those in this study.

By increasing the number of sputum specimens from 3 to 6 before the initiation of treatment for MAC, the cumulative diagnostic yield was increased from 16.5 to 23.7%. In patients with MAC-PD, bronchoscopy is often performed when sputum specimens do not aid in diagnosis. Some studies have reported the usefulness of bronchoscopy for the diagnosis of MAC-PD [16–19]. Bronchoscopy is generally relatively safe, but associated be performed are limited particularly in resource-limited areas, hence diagnosis using sputum specimens should be applied more frequently, if possible. Increasing the number of sputum specimens from 3 to 6 increased the cumulative diagnostic yield by 7.2% but an increase from 6 to 10 only increased the yield by 1.5%. There was no significant difference, but 50% of the 10 patients diagnosed using 6 sputum specimens had 1 positive culture in the first 3 sputum specimens (50% vs. 18.4%; *p* = 0.094) (Additional file 2: Table S2). Thus, for patients with at least 1 positive culture from 3 sputum specimens, it may be plausible to increase the number to 6 to facilitate a diagnosis of MAC-PD.

According to the new diagnostic criteria “ ≥ 1 positive sputum culture and GPL-core serum IgA positivity” previously proposed by Kawasaki et al. [6], now added to the conventional ATS/ERS/ESCMID/IDSA criteria [3], the diagnostic yield was 34.5% (48/139) using 3 sputum specimens. While “loose” diagnostic criteria may unnecessarily expose patients to harmful and expensive treatments, overly stringent diagnostic criteria may hinder diagnosis and delay initiation of treatment. NTM isolates including MAC have been identified in patients with chronic lung disease [20, 21]. Furthermore, MAC is an environmental commensal therefore the possibility of clinical specimen contamination cannot be excluded. In a study of 118 men in a gold-mining workforce in South Africa from whom NTM isolates were detected, only 27% met the ATS case definitions for pulmonary NTM disease [22]. Thus, the significance of a single positive culture in sputum specimens is unclear. The GPL-core IgA antibody serodiagnostic test was developed by Kitada et al. for diagnosing MAC-PD. They further assessed its accuracy by comparing with bronchial wash culture results; sensitivity and specificity were 78.6% and 96.4%, respectively [23]. A meta-analysis of the GPL-core serum IgA kit reported summary sensitivity and specificity estimates of 69.6% (95% CI, 62.1 to 76.1) and 90.6% (95% CI, 83.6 to 95.1), respectively (cutoff value, 0.7 U/mL) [24]. Considering the disease specificity of GPL-core serum IgA, chest CT findings consistent with MAC-PD, GPL-core serum IgA positivity, and 1 positive sputum culture might be sufficient criteria for the definitive diagnosis of MAC-PD. Kawasaki et al. reported that 95.5% (640/670) patients met the criteria of “1 positive sputum culture and GPL-core serum IgA positivity” and subsequently satisfied the ATS/ERS/ESCMID/IDSA guidelines diagnostic criteria for MAC-PD [6]. In this study, 6 of the 72 patients with “GPL-core serum IgA positivity and no definitive diagnosis” (Additional file 1: Table S1) and 25 of the 139 diagnosed patients met this criterion; the final diagnostic yield of this criterion was 80.1% (25/31). However, because 6 non-diagnosed patients did not undergo bronchoscopy, it is possible that the diagnostic yield would increase if bronchoscopy were performed.

This study had several limitations. Firstly, it was a single-center study in a small number of patients. Thus, the findings may not be generalizable to a larger, more diverse population. Secondly, bronchoscopy was not performed for all participants, therefore the overall diagnostic yield was possibly overestimated. Thirdly, after the first 3 sputum specimens the possibility that of disease progression over time to culture positive specifically in subsequent sputum specimens cannot be excluded. Fourthly, there was likely selection bias because some patients were diagnosed using bronchoscopy, and treatment was introduced without subsequent sputum examinations, further, there were no pre-established criteria indicating the need to perform a bronchoscopy. Fifth, diagnostic yield with 3 sputum specimens may be less than that for NTM-PD patients suspected based on chest radiograph because this study included patients with suspected NTM-PD by chest CT findings and may include those with mild clinical features also. All patient’s data generated or analyzed during this study showed in supplementary material files (Additional file 3: Table S3).

## Conclusion

The diagnostic yield for MAC-PD in 3 sputum specimens was 16.5% (23/139). Patients diagnosed using 3 sputum specimens had more severe chest CT findings including cavitary lesions. Increasing the number of sputum specimens to 6 improved diagnostic yield by 7.2%. In MAC-PD, increasing the number of sputum specimens to 6 is plausible for a definitive diagnosis.

## Supplementary Information

**Additional file 1.** **Supplement Table 1.** Clinical characteristics of patients in the diagnosis and non-diagnosis + positive GPL-core IgA groups.

**Additional file 2.** **Supplement Table 2.** Clinical characteristics of patients for whom up to 6 sputum specimens led to diagnosis.

**Additional file 3.** **Supplement Table 3.** Detailed data of all patients.

## Data Availability

All data generated or analyzed during this study are included in this article and its Additional file files (Additional file 3: Table S3). Further enquiries can be directed to the corresponding author.

## Abbreviations

AFB: Acid-fast bacilli
ATS: American Thoracic Society
BMI: Body mass index
COPD: Chronic Obstructive Pulmonary Disease
CAT: Chronic Obstructive Pulmonary Disease assessment testing
CT: Computed tomography
CI: Confidence interval
ERS: European Respiratory Society
ESCMID: European Society of Clinical Microbiology
GPL: Glycopeptidolipid
IDSA: Infectious Disease Society of America
HRCT: High-resolution CT
MAC: *Mycobacterium avium complex*
NTM: *Nontuberculous mycobacteria*
OR: Odds Ratio
PTB: Pulmonary *tuberculosis*

## References

[1] Brode SK, Daley CL, Marras TK (2014). The epidemiologic relationship between tuberculosis and non-tuberculous mycobacterial disease: a systematic review. Int J Tuberc Lung Dis.

[2] Namkoong H, Kurashima A, Morimoto K, Hoshino Y, Hasegawa N, Ato M (2016). Epidemiology of pulmonary nontuberculous mycobacterial disease. Japan Emerg Infect Dis.

[3] Daley CL, Iaccarino JM, Lange C, Cambau E, Wallace RJ, Andrejak C (2020). Treatment of nontuberculous mycobacterial pulmonary disease: an official ATS/ERS/ESCMID/IDSA clinical practice guideline. Clin Infect Dis.

[4] Tsukamura M (1991). Diagnosis of disease caused by *Mycobacterium avium* complex. Chest.

[5] Lewinsohn DM, Leonard MK, LoBue PA, Cohn DL, Daley CL, Desmond E (2017). Official American Thoracic Society/Infectious Diseases Society of America/Centers for Disease Control and Prevention Clinical Practice Guidelines: Diagnosis of Tuberculosis in Adults and Children. Clin Infect Dis.

[6] Kawasaki T, Kitada S, Fukushima K, Akiba E, Haduki K, Saito H (2022). The diagnosis of nontuberculous mycobacterial pulmonary disease by single bacterial isolation plus anti-GPL-core IgA antibody. Microbiol Spectr..

[7] Jones PW, Tabberer M, Chen WH (2011). Creating scenarios of the impact of COPD and their relationship to COPD Assessment Test (CAT™) scores. BMC Pulm Med.

[8] Morimoto K, Yoshiyama T, Kurashima A, Sasaki Y, Hoshino Y, Yoshimori K (2014). Nutritional indicators are correlated with the radiological severity score in patients with *Mycobacterium avium* complex pulmonary disease: a cross-sectional study. Intern Med.

[9] Urabe N, Sakamoto S, Ito A, Sekiguchi R, Shimanuki Y, Kanokogi T (2021). Bronchial brushing and diagnosis of pulmonary nontuberculous mycobacteria infection. Respiration.

[10] Kitada S, Maekura R, Toyoshima N, Fujiwara N, Yano I, Ogura T (2002). Serodiagnosis of pulmonary disease due to *Mycobacterium avium* complex with an enzyme immunoassay that uses a mixture of glycopeptidolipid antigens. Clin Infect Dis.

[11] Al Zahrani K, Al Jahdali H, Poirier L, René P, Menzies D (2001). Yield of smear, culture and amplification tests from repeated sputum induction for the diagnosis of pulmonary tuberculosis. Int J Tuberc Lung Dis.

[12] Marras TK, Wallace RJ, Koth LL, Stulbarg MS, Cowl CT, Daley CL (2005). Hypersensitivity pneumonitis reaction to *Mycobacterium avium* in household water. Chest.

[13] Tzou CL, Dirac MA, Becker AL, Beck NK, Weigel KM, Meschke JS (2020). Association between *Mycobacterium avium* complex pulmonary disease and mycobacteria in home water and soil. Ann Am Thorac Soc.

[14] Tamura A, Muraki K, Shimada M, Suzuki J, Kashizaki F, Matsui Y (2008). Usefulness of bronchofiberscopy for the diagnosis of pulmonary non-tuberculous mycobacteriosis—an analysis mainly on pulmonary *M. avium* complex disease. Kekkaku.

[15] Maekawa K, Naka M, Shuto S, Harada Y, Ikegami Y (2017). The characteristics of patients with pulmonary *Mycobacterium avium*-intracellulare complex disease diagnosed by bronchial lavage culture compared to those diagnosed by sputum culture. J Infect Chemother.

[16] Tanaka E, Amitani R, Niimi A, Suzuki K, Murayama T, Kuze F (1997). Yield of computed tomography and bronchoscopy for the diagnosis of *Mycobacterium avium* complex pulmonary disease. Am J Respir Crit Care Med.

[17] Ikedo Y (2001). The significance of bronchoscopy for the diagnosis of *Mycobacterium avium* complex (MAC) pulmonary disease. Kurume Med J.

[18] Sugihara E, Hirota N, Niizeki T, Tanaka R, Nagafuchi M, Koyanagi T (2003). Usefulness of bronchial lavage for the diagnosis of pulmonary disease caused by *Mycobacterium avium*-intracellulare complex (MAC) infection. J Infect Chemother.

[19] Urabe N, Sakamoto S, Sano G, Ito A, Homma S (2018). Characteristics of patients with bronchoscopy-diagnosed pulmonary *Mycobacterium avium* complex infection. J Infect Chemother.

[20] O'Brien RJ, Geiter LJ, Snider DE (1987). The epidemiology of nontuberculous mycobacterial diseases in the United States. Results from a national survey. Am Rev Respir Dis.

[21] Zhang Y, Yakrus MA, Graviss EA, Williams-Bouyer N, Turenne C, Kabani A (2004). Pulsed-field gel electrophoresis study of *Mycobacterium abscessus* isolates previously affected by DNA degradation. J Clin Microbiol.

[22] Corbett EL, Blumberg L, Churchyard GJ, Moloi N, Mallory K, Clayton T (1999). Nontuberculous mycobacteria: defining disease in a prospective cohort of South African miners. Am J Respir Crit Care Med.

[23] Kitada S, Kobayashi K, Nishiuchi Y, Fushitani K, Yoshimura K, Tateishi Y (2010). Serodiagnosis of pulmonary disease due to *Mycobacterium avium* complex proven by bronchial wash culture. Chest.

[24] Shibata Y, Horita N, Yamamoto M, Tsukahara T, Nagakura H, Tashiro K (2016). Diagnostic test accuracy of anti-glycopeptidolipid-core IgA antibodies for *Mycobacterium avium* complex pulmonary disease: systematic review and meta-analysis. Sci Rep.

